# Animal affect, welfare and the Bayesian brain

**DOI:** 10.1017/awf.2024.44

**Published:** 2024-10-08

**Authors:** Benjamin Lecorps, Daniel Weary

**Affiliations:** 1 Bristol Veterinary School, University of Bristol, Bristol BS40 5DU, UK; 2 Animal Welfare Program, Faculty of Land and Food Systems, 2357 Main Mall, University of British Columbia, Vancouver BC V6T 1Z6, Canada

**Keywords:** animal behaviour, animal welfare, emotions, predictive brain, suffering, animal cognition

## Abstract

According to the Bayesian brain hypothesis, the brain can be viewed as a predictive machine, such that predictions (or expectations) affect how sensory inputs are integrated. This means that in many cases, affective responses may depend more on the subject’s perception of the experience (driven by expectations built on past experiences) rather than on the situation itself. Little research to date has applied this concept to affective states in animals. The aim of this paper is to explore how the Bayesian brain hypothesis can be used to understand the affective experiences of animals and to develop a basis for novel predictions regarding animal welfare. Drawing from the literature illustrating how predictive processes are important to human well-being, and are often impaired in affective disorders, we explore whether the Bayesian brain theories may help understanding animals’ affective responses and whether deficits in predictive processes may lead to previously unconsidered welfare consequences. We conclude that considering animals as predictive entities can improve our understanding of their affective responses, with implications for basic research and for how to provide animals a better life.

## Introduction

Emotions are often conceptualised as responses to a specific situation. Under this conception, it is often assumed that animals simply respond in accordance to a situation’s characteristics, similarly to how symptoms were once believed to directly reflect a physiological dysfunction (Ongaro & Kaptchuk 2019). However, many studies have shown that the animal’s perception of the stressor can sometimes better explain their emotional response than can the objective characteristics of the situation (e.g. Weiss 1972).

The Bayesian brain hypothesis (also referred to as the predictive brain or predictive mind) states that an individual does not respond ‘naïvely’ to new information (arising from senses); rather, this new information is integrated within the context of expectations (termed ‘priors’) generated from past experiences (Box 1). This effect can be illustrated by a range of perceptual illusions, such as the size-weight illusion where a smaller object is rated as heavier than a larger one despite being of similar weight (Ellis & Lederman 1993). Cognitive explanations for this illusion involve the expectation that the bigger object should be heavier than the smaller one; when this is not confirmed by sensory inputs people evaluate the smaller object as heavier. This type of illusion may come from innate predisposition, can involve the application of general rules of physics learnt through day-to-day interactions (e.g. bigger objects are typically heavier), or be affected by specific experiences. For instance, golfers who know practice balls are heavier than real golf balls will erroneously judge different types of balls (of equal weight) as different while people unaware of this difference will judge them to be similar (Ellis & Lederman 1998). It is important to note that ‘expectations’ do not imply conscious, explicit knowledge but can also refer to implicit information (Box 2).

**Box 1. The Bayesian brain hypothesis.** The term ‘Bayesian brain’ is commonly used in cognitive sciences but may be misleading to some. As Friston (2012) explains, the idea is simply that “*we are trying to infer the causes of our sensations based on a generative model of the world*”. Predictive theories do not seek to replace a bottom-up approach with a top-down one, but rather provide an explanation for how the brain integrates information coming from senses (interoceptive and exteroceptive) in a context that is set by priors (largely determined by past experiences). These theories stem from Friston’s generative models specifying both the prior distribution of causes and the likelihood of input given the causes (Friston 2010).

**Box 2. Implicit vs explicit expectations.** How perception is affected by implicit or explicit knowledge has been explored in a variety of contexts. People can use both explicit and implicit knowledge, but it appears that implicit knowledge, acquired through day-to-day interactions with the world, is often used to predict events encountered through life. For instance, most people have a limited understanding of physics when asked to express this verbally (Vicovaro 2021), but they can show an accurate implicit understanding (Kozhevnikov & Hegarty 2001); for example, an implicit understanding of motion allows people to determine the trajectory of moving objects and to intercept them. Illusions driven by implicit expectations are often ‘cognitively impenetrable’. For instance, the conscious knowledge that the two objects are of similar weight does not reduce the perceptual illusion that the smaller one seems heavier (Flournoy 1894). ‘Prior beliefs’ in the context of Bayesian processing often can be assumed to be implicit (Van den Bergh *et al.* 2021) and can therefore be explored without implying conscious evaluations.

If expectations can trick us into believing that two objects are not of the same weight, can they also affect our emotions? Research on human pain illustrates how the shift towards a Bayesian perspective allows a better understanding of emotional responses (e.g. Hoskin *et al.* 2019). In this field, the role of expectations in modulating pain responses is well recognised. A variety of studies have shown that beliefs about pain affect the perception, response, and recovery from painful experiences, such that pain is now conceived as a combination of top-down and bottom-up mechanisms (Wiech & Shriver 2018). In particular, pain experiences are seen to be affected by *“the sufferer’s beliefs about pain*” (Hoskin *et al.* 2019), as illustrated by placebo and nocebo effects (that either minimise or exaggerate pain expectations, respectively) (Stewart-Williams & Podd 2004).

Many empirical studies show that expectations can have a substantial effect on affective responses to pain. For example, a negative expectation can abolish the analgesic properties of a drug (e.g. Remifentanil), whereas a positive expectation can double its effect (Bingel *et al.* 2011). These findings are backed by neurobiological evidence showing that higher-order brain regions can affect lower-order ones to suppress or enhance the mismatch between expectations and perception (Schenk *et al.* 2017; Jepma *et al.* 2018).

**Figure 1.**
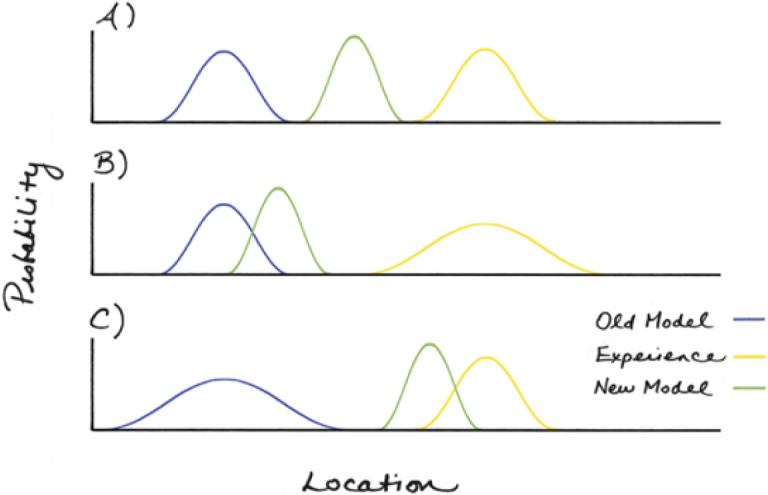
A graphical representation of how expectations resulting from prior experiences (also termed ‘priors’) are affected by new sensory experiences. Panel A illustrates how the new model (illustrated in green) is a result of animal’s current sensory experiences (shown in yellow) and its former expectations (or priors, shown in blue). The extent to which the new model is updated is affected by the confidence (also termed precision) in expectations (as illustrated in Panel B, where high confidence in expectations results in minor modification to the new model) and by precision of the current sensory experience (shown in Panel C), where high confidence in current sensory inputs results in substantial modification to the new model. Artwork by Ann Sanderson. Figure redrawn from Pezzulo *et al.* (2019).

Although expectations can bias perception, sensory inputs are still attended to. The Bayesian brain hypothesis does not take a purely top-down approach, rather it considers that sensory information is computed within the context of the prior knowledge, such that “*a single highly flexible inferential process fluidly and constantly combines top-down predictions with bottom-up sensory information*” (Nave *et al.* 2020). Priors and sensory information can be viewed as probability density functions with margins of uncertainty (Figure 1). Expectations do not necessarily take precedence and may be discarded if they fail to predict current sensory experiences (see Figure 1C).

The weight given to sensory inputs is affected by the strength of the individual’s belief. For example, in randomised controlled trials, people are typically told they have a 50% chance of receiving the drug. When patients are given a placebo but told that they were administrated an analgesic, their pain threshold is typically much higher than people who were told they had a 50% chance of receiving the analgesic (Rief & Glombiewski 2012). Expectations of receiving a treatment can be artificially strengthened (via sensory inputs) by pairing a placebo with an agent that can be directly sensed by participants (e.g. has a distinctive prickling sensation but no analgesic effect); these participants are more likely to respond as if they had received an analgesic compared to participants who were not provided the perceptual cue (Rief & Glombiewski 2012). These findings among others (Hoskin *et al.* 2019), suggest that the affective response to pain is affected by expectations and that the effect of expectations is dependent on the patient’s certainty. As a rule of thumb, the lower the certainty, the more weight is placed on sensory inputs (Tabor & Burr 2019).

Updating expectations is another important feature of the Bayesian brain theories. If expectations were fixed, individuals would not be able to fine tune their responses to a changing environment. Thus, individuals are thought to use new information to refine predictions for future experiences, so as to reduce discrepancies between expectations and experiences (termed ‘prediction errors’). For instance, subjecting a patient to a high-intensity stimulus when they expected to receive an analgesic may lead to the belief that the analgesic is ineffective (Büchel *et al.* 2014) and this new belief can then inform expectations for future experiences. The extent to which new information results in changing expectations is based on confidence in both the new data and the existing model.

As of now, we have mostly taken examples from the human pain literature because we believe it provides a relevant starting point. However, it should be noted that the Bayesian brain application is not limited to pain (Box 3), or to humans. In the sections that follow, we provide an overview of how the Bayesian brain hypothesis can help understanding affective experiences in animals. Expectations are too rarely (or not explicitly) considered when studying affective states in animals and we argue that the Bayesian brain hypothesis can help refine our understanding of these experiences. To illustrate the usefulness of this new perspective, we first explore how it has already been applied in the field of animal cognition, before exploring how it might now be applied to understanding animal affective responses. In the final section, we explore how a Bayesian view can also help understand animal welfare in a way that is consistent with perspectives on human well-being (Miller *et al.* 2022). For example, we discuss how mood-related disorders in humans often involve issues with updating processes (e.g. patients display persistent negative beliefs), and we call for research on dysfunctions in updating processes in animals, an issue we term ‘Bayesian blindness’.

**Box 3. Other affective experiences.** The Bayesian brain hypothesis can help explain complex integrations of interoceptive and exteroceptive signals for a range of feelings, for example, “*why the sensation of a fast-beating heart feels so different when we suspect heart failure than it does after (say) a bout of vigorous exercise*” (Nave *et al.* 2020). Expectations alter results obtained via randomised controlled trials for a range of experiences (Finniss *et al.* 2010). For instance, participants told they had just received caffeine showed higher cardiovascular activity than those told they had a 50% chance of receiving the drug (Kirsch & Weixel 1988). Research has now started using predictive theories to better understand mechanisms driving complex social behaviours (Brown & Brüne 2012), arguing that to thrive in social environments one must be a good predictor of others’ actions and intentions. Predictive processes are likely to play a role in many affective experiences and likely other cognitive processes.

## A Bayesian approach to learning and affect in animals

Understanding the affective experiences of animals remains a major challenge and an essential endeavour to improving the welfare of animals. Assessing expectations in non-verbal subjects can be challenging, but different experimental paradigms provide direct and indirect approaches, mainly by looking at how animals make decisions. Here, we will first explore the evidence suggesting that animals do behave (or at least learn) as Bayesian entities before exploring how this may alter their affective responses.

### A Bayesian conception of animal learning

Animal learning theories have long adopted Bayesian models, progressively replacing traditional ‘associative learning models’ derived from the Rescorla-Wagner (1972) model. The main difference between traditional and Bayesian models is that the former assumes that, at any given time, an animal’s knowledge is represented by a single value (or associative weight) whereas the latter assumes animals will “*entertain a spectrum of hypothetical values*” (Kruschke 2008). Thus, Bayesian models represent uncertainties in an animal’s belief (often represented as a distribution, with a greater spread reflecting lower certainty). Bayesian models of animal learning assume that when new information is processed, it will strengthen or weaken the individual’s belief in specific hypotheses. For instance, if human-animal interactions are increasingly negative, the belief that humans are ‘bad’ will strengthen and the belief that humans are good will shrink. The implication is that “*Bayesian models generate probabilistic rather than deterministic anticipations*” (Kruschke 2008). This matters because it means the brain is often ready to see more than one hypothesis and some relatively likely alternatives are more strongly predicted than less likely ones even though they are not the ‘dominant’ prediction.

Bayesian models provide a more sophisticated explanation for multiple forms of learning and help explain many learning-related phenomena such as overshadowing, backward blocking and latent inhibition (for a review, see Courville *et al.* 2006). Bayesian models can also help explain why animals learn faster when surprised (e.g. Kamin 1967), as these models incorporate how prediction errors affect learning (Courville *et al.* 2006). It is beyond the scope of this paper to provide an in-depth review of the many advantages of considering animals as “*Bayesian learners*” (Gershman 2015), but the advantages highlighted by animal learning theorists may also apply to the study of animal emotions.

### Influence of past experiences on the affective responses of animals

Some experimental designs in the animal emotion literature are based on the idea that animals learn from past experiences, and that this knowledge can influence affective responses. One key example is fear conditioning. Before conditioning, the animal is expected to show little response to a neutral stimulus such as an audible tone. However, if this tone is repeatedly paired with a negative stimulus, like an electric shock, the animal will begin to respond negatively to the tone. Fear conditioning is best explained by probabilistic inference (Tzovara *et al.* 2018) and may involve both implicit and explicit expectations (Benedetti 2020), as evidenced by experiments showing that temporal contiguity between the conditioned and unconditioned stimuli is not enough to elicit fear-conditioned responses (Kamin 1967). Furthermore, some experiments with animals have shown ‘trace conditioning’, a variant of classical conditioning where the time between the unconditioned stimulus (a predicting cue) and the conditioned stimulus (e.g. a reward) progressively increases (Clark & Squire 1998). For instance, hens could be trained to associate a cue with a reward despite a trace of up to 22 s, as illustrated by anticipatory behaviours displayed between the CS and US presentations (Moe *et al.* 2009); these results illustrate one approach to measuring expectations in animals.

Animals can make sophisticated trade-offs between rewards and punishments (e.g. Rushen 1996). In one study from our research group, Ede *et al.* (2018) showed that dairy calves would accept a painful experience (caused by an intramuscular injection) if they expected a substantial reward (1 L of milk), but fewer calves would do so when they learned that less milk would be provided. Calves trained to expect a less painful subcutaneous injection were willing to accept this even when the smaller reward was expected. This example illustrates how animals can make trade-offs based on expectations regarding both how much pain they will experience and how much reward they will receive. This thinking is implicit in most consumer demand studies where animals learn to expect a specific reward for their work and trade-off that work for an expected outcome (e.g. von Keyserlingk *et al.* 2017; Wenker *et al.* 2020). In paradigms aiming to assess impulse control, animals may also be asked to make a choice between a higher valued but delayed reward versus an immediate reward of lesser value. For instance, Zebunke *et al.* (2018) showed that piglets could learn to wait for up to 24 s when expecting to receive a better-quality reward, showing that animals can frame expectations between two options as a function of time.

Research on stress responses in animals has also emphasised the importance of expectations (for a review, see Koolhaas *et al.* 2010). In a well-known study, rats (*Rattus norvegicus*) given the ability to predict the occurrence of electrical shocks suffered less from stress-related disease compared to those that could not predict when shocks would occur, although both groups received the same number of shocks (Weiss 1972). This result highlights that shocks alone do not account for the risk of stress-related disease. Unpredictability is now commonly seen as worsening the effect of an aversive situation, and something animals will choose to avoid if they can (Bassett & Buchanan-Smith 2007). Unpredictable housing (Harding *et al.* 2004) or unpredictable exposure to stressors (Doyle *et al.* 2011) can lead to negative mood.

Providing animals with additional knowledge about the stressor (e.g. magnitude, duration) and control allows animals to act on this knowledge, for example, by changing their behaviour in ways that allow them to minimise exposure to the stressor. For instance, rats given control over shocks (by pressing a lever to make them stop) were at lower risk of developing stress-related pathologies than rats that received the same number of shocks but could not control their occurrence (Weiss 1972). Similarly, sheep that were trained to respond to cues to avoid electric shocks (delivered via a virtual fence) did not show elevated stress responses (Kearton *et al.* 2020). Perceived control allows individuals to benefit from a type of placebo effect, reducing the expectation and experience of unpleasant affect.

Seeing learnt responses from a Bayesian perspective may help refine experimental approaches. Studies often provide animals with a limited number of experiences from which to gain new knowledge. In the case of push-gate studies, where weight is typically increased over time, animals need to continuously update their expectations regarding how much work is required. As prior updating is probabilistic, animals would likely benefit from multiple exposures to update their previously established model, perhaps even training animals to expect variation in weight (rather than a specific weight); in this way animals should build a model less affected by the previous weight that they encountered and thus be more prone to continue trying. Previous work has used partial (Foster *et al.* 1997) or variable (de Jonge *et al.* 2008a) reinforcement schedules to reduce the risk that future behaviours are highly influenced by recent experiences, sometimes as a means to slow down learning (e.g. Neave *et al.* 2013). The Bayesian view also pushes us to consider elements that are rarely considered, such as the animal’s confidence in their expectations, their ability to collect information about the situation, the predicted consequences, and whether the animal expects to be able to cope with the situation. All of these aspects, which refer to how animals seek and process knowledge, require attention in future studies as they may change the way a situation is experienced. Although, some meta-cognitive aspects (e.g. how confident animals are in their expectations) may prove hard to explore, other aspects (e.g. the effect of knowledge about the stressor) may be relatively easy to manipulate and assess.

### A Bayesian view can help understanding otherwise counter-intuitive responses in animals

#### Placebo effects

Placebo and nocebo effects can be explained when the role of expectations is considered, and several studies on non-human animals have shown evidence of these effects. In one example, Herrnstein (1962) illustrated that similar changes in behaviour were observed in rats injected with scopolamine (a drug that negatively affects learning) as in rats injected with a saline solution after experiencing scopolamine injections. In another study, Nolan *et al.* (2012) trained rats to tolerate non-injurious thermal pain to access a sweet solution. Morphine injections increased success in accessing the sweet solution, as expected given the analgesic effects of morphine. However, this effect persisted even when morphine was later replaced by a placebo, suggesting that the expectation of receiving an analgesic reduced the affective response to thermal pain. Interestingly, rats with higher sensitivity to morphine were more placebo sensitive, a result that mirrors findings in humans (Amanzio & Benedetti 1999). Future studies should further explore whether animals experience placebo and nocebo effects in both pain and non-pain contexts. As we will explore, engineering positive expectations may be a powerful way to mitigate the effect of stressors.

#### Surprise

We have briefly touched on how surprise (or prediction error) accelerates learning. In humans, prediction errors can also result in emotional responses (e.g. Hoemann *et al.* 2017; Van de Cruys & Van Dessel 2021). Similar experimental designs have been used when studying the effect of surprise on both learning and affect. These designs involve creating expectations (e.g. that food is delivered in a familiar environment) before violating them (e.g. by suddenly opening an umbrella next to the animal), and then comparing responses of animals that have been surprised or not (e.g. Courville *et al.* 2006). Perhaps unsurprisingly, sudden events can trigger brief but intense responses in animals (e.g. horses; Lansade *et al.* 2008). Changes in the intensity of the reinforcer have also been used to assess emotional responses to surprise in animals. For example, animals respond positively when provided larger treats than expected (Boissy *et al.* 2007) and *vice versa* for negative contrasts (Crespi 1942). Contrast effects illustrate how prediction errors can modulate (positively or negatively) the emotional response to stimuli. However, contrast effects are not always detected in animal studies; for examples, studies by Tan *et al.* (2020) on zebrafish (*Danio rerio*) and by Davies *et al.* (2015) on chickens both failed to find these effects. These failures may be related to methodological issues or to species differences in how expectations are processed.

The reinforcing nature of positive prediction errors can help explain the results of a series of studies on pigeons (*Columba livia*) choosing between a feed source that is rewarded 50% of the time versus another that is rewarded 100% of the time (see Ajuwon *et al.* 2023). From the perspective of optimising resource gains, one would expect pigeons to prefer the always rewarded option, but instead pigeons often display no preference (Smith & Zentall 2016), or even prefer the partially rewarded option (Case & Zentall 2018). One explanation is that the intermittently rewarded option is a source of positive contrasts between the expected outcome (i.e. 50%) and the value of the reinforcement received. In other words, pigeons may prefer the 50% option because the uncertainty made each reward more rewarding. Consistent with this perspective, Zentall *et al.* (2019) found that increasing the reinforcement rate to 75% (which reduced the contrast between expectations and reinforcement) decreased the preference for the ‘suboptimal’ alternative. Together, these results suggest that prediction errors affect the perceived value of a reward and may lead animals to mistakenly believe that one option leads to a better reward.

#### The exploration paradox

Clark (2013) noted the apparent paradox between the need to reduce prediction errors and the fact that animals engage in actions that apparently increase the risk of prediction errors (e.g. they do not “*simply seek a nice dark room and stay in it*”). Considering information gathering as essential to reducing prediction errors may help understanding why animals appear motivated to explore their environment despite energetic and survival costs. Animals may especially value the opportunity to refine predictions by gathering information in circumstances where predictions are weak (e.g. in a new or rapidly changing environment; Courville *et al.* 2006).

Exploration allows animals to refine the representations they build about their world, likely driven by positive (e.g. exploration being rewarding) and negative affect (e.g. growing uncertainty leading to feelings of anxiety). This view is consistent with neurobiological evidence indicating that seeking novelty is rewarding (Franks *et al.* 2013) and can reduce feelings of anxiety associated with uncertainty (Seth & Friston 2016). Work in primates has shown that animals prefer to receive unambiguous information about the magnitude of rewards (Bromberg-Martin & Hikosaka 2009; Blanchard *et al.* 2015) and can learn to actively search for cues to reduce uncertainty (Daddaoua *et al.* 2016). This hypothesis, that information gathering is reinforcing, has also been evaluated in rats. Animals consistently preferred options where information was available, even if the information did not change the outcome of the choice (Ajuwon *et al.* 2023). However, signals for positive outcomes had a greater influence on animals’ preference, consistent with the conditioned reinforcement effect (positive cues reinforce responses more than negative ones), suggesting that both uncertainty reduction and conditioned reinforcement are involved in this process.

What drives animals to explore their environment is the subject of debate (e.g. Rushen 1993), but we suggest that Bayesian brain theories can help explain the motivation to explore because uncertainty is a core feature of these models. If animals judge their belief (e.g. that an environment is safe) as uncertain, it makes sense to actively engage in exploration to reduce this uncertainty. From this perspective, information about the environment and the associated decrease in uncertainty, is the reward for exploration, which may explain why exploration is seen as self-rewarding (Byrne 2013; Franks *et al.* 2022). The motivation to explore (or more generally to acquire information) may explain why animals perform activities that are not associated with external rewards (Huebner 2012). For example, primates will ‘work’ to solve mazes and puzzles even when they receive no external rewards for completing the task (Harlow 1950). Mice (*Mus musculus*) will even expose themselves to dangers to explore a new environment (Dashiell 1925), hungry rats will choose to explore a new environment rather than eat (Small 1899) and prey species may explore signs of predator presence despite the associated predation risks (e.g. Brown 2003; Monclús *et al.* 2006). The costs of exploration can be high; mice that were more explorative in an open field were also at higher risk of predation (Renner 1990). That animals are willing to accept such costs suggests that acquiring information is highly valued.

Similarly, the observation that animals will often work for a resource that they can otherwise get for free (‘contrafreeloading’; e.g. de Jonge *et al.* 2008b; Van Os *et al.* 2018) is likely related to the motivation of animals to understand their surroundings. Some have argued that animals may be rewarded by acting to get a reward (i.e. a feeling of agency; Inglis *et al.* 1997), but we favour the idea that these behaviours are related to an inherent motivation to maintain accurate predictions (i.e. the information gathering function; Hintze & Yee 2023) about different options as suggested by the finding that animals will access rewards using a combination of work and free-access options when given the choice (e.g. Rosenberger *et al.* 2020).

The continued use of apparently non-optimal options is often taken as evidence of poor learning ability, but such continued sampling can instead be considered as a type of information gathering (Bednekoff & Balda 1997). Continued ‘errors’ may reflect a motivation to sample even when the likelihood of reward is low, and this type of explorative sampling behaviour allows animals to update reward probabilities in a potentially changing world. Overall, evidence from exploratory behaviour, contrafreeloading, and continued sampling in learning tasks reflects the importance of Bayesian prediction errors in understanding animal behaviour and affect.

## Failures in updating expectations

Some authors contend that a “*general deficiency in prediction updating*” is core to the emergence and maintenance of affective disorders in humans (Van de Cruys & Van Dessel 2021; Van den Bergh *et al.* 2021). We suggest that this line of thinking should also stimulate research in animal welfare, with special implications for animal suffering (Weary 2019). As we shall review in the section that follows, a predictive processing perspective provides a basis for some ideas already of interest in the animal welfare literature, and in some instances leads to novel predictions with welfare implications that have not yet been explored. In this section, we review recent progress on the role of predictive processing in mental distress and psychopathologies in humans and use this to generate predictions regarding subjective well-being of captive animals.

### Persistent, generalised negative beliefs and emergence of affective disorders

Many cognitive vulnerabilities (e.g. pessimistic outlook, intolerance to uncertainty) have been linked with the risk of developing affective disorders such as depression or anxiety. Research suggests that these cognitive risk factors share a common core of dysfunctional negative beliefs (Hong & Cheung 2015; Kube & Rozenkrantz 2021). These beliefs may lead a person *“to appraise situations as threatening, to hold negative anticipations about oneself and the world, to experience negative mood states and emotions, and to show poor emotion regulation*” (Van den Bergh *et al.* 2021).

Negative beliefs may emerge when experiencing negative situations, especially if they conflict with initial predictions. Active inference theories (Friston 2010) propose that to reduce prediction error, one may attempt to either act on what it experiences to improve the fit between their existing model and their current experience or update their model (belief update) to better fit their current experiences (Barrett & Simmons 2015). What drives the use of either strategy is not well understood, but the consequences for well-being are marked. The latter process may lead to the belief that, regardless of their actions, the negative experiences will persist. This typically results in a growing lack of confidence in the efficacy of one’s actions to mitigate negative feelings and leads to the perception of being powerless, resulting in the adoption of strategies based on avoidance (e.g. social withdrawal) and to extending negative beliefs to oneself.

Generalised negative beliefs and avoidance coping mechanisms make it hard to update priors, as limited contradictory new evidence is presented to the model. For example, people will adapt to injuries by limiting movements that trigger pain. Once the pain is gone, mobility can resume. However, fear of pain can lead to people displaying protective behaviours long after the pain is gone, contributing to long-term disability (Meulders *et al.* 2017). From the perspective of the predictive brain, if one avoids moving because of the fear of pain, one cannot know that these movements are no longer painful and thus the model is not challenged by new evidence.

The nature of the predictive processing system may also push people to seek situations or evidence that confirm existing biases, producing “*a powerful feedback loop where the belief in one’s inability to reduce prediction error through action leads the agent to sample the environment for evidence of this inability, which confirms and supports the negative belief*” (Miller *et al.* 2022). In other words, people may show selective attention to confirmatory evidence, may interpret new data in a way that confirms pre-existing beliefs, and avoid information that would contradict these beliefs (Bruineberg *et al.* 2018; Kube & Rozenkrantz 2021). To our knowledge, evidence for this type of confirmation bias has yet to be explored in animals.

The maintenance of negative beliefs, and their generalisation to one’s competencies and to the future, can lead to a narrowed ability to update priors, as the individual is no longer experiencing situations that could provide contradicting evidence, or because the limited contradicting evidence experienced is rejected given that it does not fit the individual’s well-established model. Such effects are illustrated in depressed patients (Everaert *et al.* 2017) and people with anxiety disorders (Kube & Rozenkrantz 2021) who sometimes struggle to update established negative beliefs. In the section that follows, we examine the examples of psychological processes in non-human animals that may relate to failures in updating priors and thus interfere with predictive processes.

### Evidence of deficits in updating processes in non-human animals

Kube and Rozenkrantz (2021) identified three mechanisms by which people affected by different psychological disorders display reduced prior updating capacities: 1) attention precision given to prior beliefs (i.e. increased weight given to existing beliefs rather than to new information), 2) interpretation biases (a tendency to interpret new information as confirmatory), and 3) reappraisal (questioning the validity of dis-confirmatory evidence). Here, we highlight work related to re-learning deficits and persistent negative expectations in animals that show intriguing similarities with the reduced prior updating capacities seen in humans. More work is needed to explore this phenomenon in non-human animals, but any of the following markers might be used to infer about animals’ reduced ability to update their model of the world that we refer to as ‘Bayesian blindness’ – an inability to update or access expectations.

#### Re-learning deficits

Studies have demonstrated that, in certain situations, animals may experience difficulties in updating priors and lack cognitive flexibility or a capacity to re-learn (often assessed by a reversal task; e.g. if A was rewarded and B punished, then B becomes rewarded, and A punished). Some animals behave as if they are incapable of learning a new rule and instead appear to be stuck in their pre-existing model. This reduced cognitive flexibility has been observed in animals reared in suboptimal conditions, such as individually housed dairy calves (e.g. Meagher *et al.* 2015). This type of cognitive deficit may stem from any of the three processes highlighted by Kube and Rozenkrantz (2021); further work is required to determine if this arises from attention, judgment, or memory deficits.

Negative situations can trigger quick and efficient learning. For instance, animals can associate a specific environment (e.g. a distinctly coloured enclosure) or a specific cue (e.g. a light) with the experience of pain or fear (e.g. Liberzon & Knox 2012) and will then avoid these cues in the future. Although any learnt response is expected to wane once the conditioned stimulus is no longer paired with the unconditioned stimulus (i.e. after a period of extinction training), in some cases conditioned fear responses can persist far longer than expected, suggesting that the animal is unable to update its negative expectations. For instance, rats subjected to a single prolonged stressor (used in studies intending to model post-traumatic stress disorders) show deficits in fear extinction learning. They continue to display freezing behaviours (> 20% of the time) long after (up to ten days vs less than four days for controls) the environment is no longer paired with shocks (Winters *et al.* 2021). Other factors may affect fear extinction learning. For instance, mice that are susceptible to social defeats (i.e. they quickly display prolonged social avoidance) also show extended fear learning (Chou *et al.* 2014). Thus, some animals appear to be prone to developing beliefs about fear-eliciting situations and to maintain these expectations; this individual propensity may be related to an earlier trauma or stem from stable individual differences.

Learned helplessness provides another example of a learned response that is resistant to extinction. After repeated experiences with an unescapable threat, some animals will stop responding (e.g. they will no longer show escape attempts). To some extent, this response can be mistaken for habituation, as in both cases the behavioural response wanes to what was initially an aversive experience. The crucial difference is that, in the case of habituation, the reduced response reflects that the stimulus is no longer experienced as aversive, while in the case of learned helplessness, the reduced response is due to the animal no longer perceiving its response to be effective. Helpless rats show a reduction in reward sensitivity, a higher susceptibility to conditioned fear, and a deficit in fear extinction (Shumake *et al.* 2005). This phenotype suggests that helpless animals display some characteristics consistent with deficits in updating processes, perhaps linked to deficits in reappraisal (given that drugs promoting memorisation typically improve extinction learning in helpless rats; Wrubel *et al.* 2007). We suggest that learned helplessness illustrates how encountering repeated negative affective experiences can lead to negative beliefs about animals’ own competencies.

These examples show how animals can sometimes appear to be stuck in the past and unable to learn from new experiences, and suggest that this cognitive deficit can be associated with early-life trauma, lack of cognitive stimulations, and persistent exposure to negative events. We predict that animals exposed to such conditions are more likely to experience deficits in their ability to update expectations, negatively affecting both their current welfare and their future capacity to cope with new events. Until now, only limited research has explored how cognitive flexibility, including the capacity to update priors, improves animals’ coping abilities.

#### Persistent negative expectations

The term fearfulness is often used in the animal welfare literature to describe a condition that may be similar to persistent anxiety in humans. Animals are described as fearful if they are averse to novelty (e.g. show reduced exploration of novel stimuli) and are highly reactive to stimuli perceived as threatening. The factors that lead animals to become fearful and the lifelong implications of fearfulness are poorly understood. Absence of maternal care (in chicks) or early social isolation (in dairy calves) are examples of environmental factors that lead to increased fearfulness (Meagher *et al.* 2015; Edgar *et al.* 2016). Persistent fearfulness may be due to, or lead to, deficits in predictive processing, similarly to what has been suggested for people exhibiting signs of anxiety disorders or personality traits, such as pessimism and neuroticism (Carver & Scheier 2014; Van den Bergh *et al.* 2021). How animals respond to new or threatening situations can be detected early in life and, in some individuals, remains stable over time (De Haas *et al.* 2012; Lecorps *et al.* 2018b; Christensen *et al.* 2020; Neave *et al.* 2020). Fearfulness has received some attention in the animal welfare literature, but mostly as an outcome (e.g. poor conditions lead to heightened fearfulness). New work is required on how fearfulness develops and how it affects the way animals cope with challenges. We suggest that fearfulness in animals may lead to persistent updating issues, to the extent that this reduces the animal’s exposure to situations that can contradict their beliefs.

Stressors can also generate negative expectations about unrelated situations. For example, the same rats that displayed diminished fear extinction following a single-prolonged stressor also showed fear overgeneralisation (Winters *et al.* 2021), meaning that non-threatening new environments also triggered fear responses. Accumulating evidence suggest that stressors impact how animals interpret ambiguous information, with a shift towards greater pessimism. Unpredictable housing (Harding *et al.* 2004) and pain (Neave *et al.* 2013), among many other experiences (for a review, see Lagisz *et al.* 2020; Neville *et al.* 2020), can induce negative perceptions of otherwise ambiguous situations. Long-term changes in judgment bias in response to stressors have rarely been explored (e.g. Paul *et al.* 2023), but persistent pessimism (as a trait) has been observed in several species and is thought to make animals more vulnerable to stressors (for a review, see Lecorps *et al.* 2021a) with examples in rats (e.g. Rygula *et al.* 2013), dairy calves (Lecorps *et al.* 2018a, 2020) and pigs (Horback & Parsons 2019).

There is a rich history of animal research on persistent changes in anxiety-related and depressive-like behaviours that involve persistent negative expectations such as anhedonia (the apparent de-valuation of rewards), typically in response to sub-optimal housing or chronic exposure to uncontrollable stressors (Wang *et al.* 2017). Such behaviours are consistent with generalised negative expectations about potential threats (such that the animal tends to overestimate the negative consequences) and underestimation of potential rewards (the likelihood/value of rewards). The latter is particularly interesting given that, despite consuming some sucrose water, anhedonic animals no longer prefer this to water (e.g. Rygula *et al.* 2005). These results suggest that some top-down mechanisms downplay the rewarding property of sucrose or the ability to remember this as rewarding. Similarly, it has been reported that patients with major depressive disorder will enjoy accessible rewards but display no motivation to access these (Treadway & Zald 2011), suggesting that these patients display deficits in prior precision such that they do not perceive a treat to be rewarding until it is consumed. Persistent anhedonia-like responses have been shown in a variety of species living in stressful conditions (e.g. pigs; Figueroa *et al.* 2015) or after potentially traumatic experiences (e.g. in dairy cows; Lecorps *et al.* 2021b), but these responses remain poorly understood. Future work should explore whether anhedonic animals display a generalised and persistent devaluation of any rewarding activities and whether this state is associated with perceptual, appraisal, or memorisation deficits.

## Limitations

We have reviewed some of the literature that suggest animals act as Bayesian entities and attempted to illustrate how this could affect their affective responses and welfare, but there are several limitations to our conceptual review. First, we have looked at evidence illustrating key aspects of the application of this perspective to animal affect, including examples that are difficult to understand when using a bottom-up view of affective experiences. These examples were selected based on what we judged to be their relevance, as in any narrative review. The examples provide evidence of how a Bayesian perspective can prove useful, but our approach should not be seen as a definitive test. We conclude simply that Bayesian brain theories can usefully generate hypotheses, and that future tests of these hypotheses may lead to an improved understanding of animal affective responses.

Second, although the application of the Bayesian theorem is recognised as one of the most influential ideas in cognitive sciences (Melnikoff & Strohminger 2024), some areas of human cognition, such as belief polarisation (e.g. when a person strengthens their belief despite strong evidence that it is wrong), appear inconsistent with this theorem (Mandelbaum *et al.* 2020). Our goal is not to convince readers that animals must always be considered Bayesian entities, but rather that Bayesian theories can help generate useful predictions and insights regarding what they feel.

Third, we have deliberately taken a generic stance in applying the concepts discussed to ‘animals’; we acknowledge that species are likely to differ in the way they process information and use predictive processes, but argue that in many cases understanding the predictive element will allow for an improved understanding of affective states and welfare. Whether some species process information in a non-Bayesian way remains to be explored.

## Animal welfare implications and conclusion

The Bayesian brain hypothesis has led to new insights regarding human cognition, affect and well-being. We reviewed evidence suggesting that these theories can be applied to non-human animals and explored how they can help understand affective responses in animals. Animals are predicted to react to situations in accordance with their past experiences; understanding how past experiences can affect beliefs is thus of broad importance to work exploring how animals respond to stressors, a key aspect of many welfare studies. Moreover, we point to the possibility that deficits in an animal’s ability to update or access their beliefs (Bayesian blindness) can result in persistent and broad negative welfare impacts, as shown in humans. We urge new work to focus attention on the contextual factors that explain why animals react the way they do, as well as more applied research seeking to prevent the formation and maintenance of negative beliefs. As an example of the latter, work is required on how best to engineer more positive expectations, such as those that can be achieved via positive reinforcement training, shifting the animals’ perception of mildly aversive stimuli to neutral or even positive. We also encourage new basic research on the factors that interfere with the development of expectations in animals, including housing and management practices that diminish learning or otherwise undermine the animal’s ability to develop an accurate model of their world and the efficacy of their actions.
